# Bayesian‐optimized deep learning for identifying essential genes of mitophagy and fostering therapies to combat drug resistance in human cancers

**DOI:** 10.1111/jcmm.18254

**Published:** 2025-01-21

**Authors:** Wenyi Jin, Junwen Chen, Zhongyi Li, Zhang Yubiao, Hao Peng

**Affiliations:** ^1^ Department of Orthopedics Renmin Hospital of Wuhan University Wuhan China; ^2^ Department of Biomedical Sciences City University of Hong Kong Hong Kong China; ^3^ Department of General, Visceral and Transplant Surgery Ludwig‐Maximilians‐University Munich Munich Germany

**Keywords:** Bayesian optimization, CERS1, deep learning, mitophagy

## Abstract

Dysregulated mitophagy is essential for mitochondrial quality control within human cancers. However, identifying hub genes regulating mitophagy and developing mitophagy‐based treatments to combat drug resistance remains challenging. Herein, BayeDEM (Bayesian‐optimized Deep learning for identifying Essential genes of Mitophagy) was proposed for such a task. After Bayesian optimization, BayeDEM demonstrated its excellent performance in identifying critical genes regulating mitophagy of osteosarcoma (area under curve [AUC] of ROC: 98.96%; AUC of PR curve: 100%). CERS1 was identified as the most essential gene regulating mitophagy (mean (|SHAP value|): 4.14). Inhibition of CERS1 sensitized cisplatin‐resistant osteosarcoma cells to cisplatin, restricting their growth, proliferation, invasion, migration and colony formation and inducing apoptosis. Mechanistically, inhibition of CERS1 restricted mitophagy to destroy the mitochondrial quality control in cisplatin‐resistant osteosarcoma cells, including mitochondrial membrane potential loss and unfavourable mitochondrial dynamics, rendering them susceptible to cisplatin‐induced apoptosis. More importantly, mitophagy facilitated the immunosuppressive microenvironment formation by significantly modulating T‐cell differentiation, adhesion and antigen presentation, and mitophagy mainly affects malignant osteoblasts in the early‐mid developmental stage. Immunologically, mitophagy potentially modulated the MIF signalling transmission between malignant osteoblasts and B cells, DCs, CD8+ T cells, NK cells and monocytes through the MIF‐(CD74 + CXCR4) receptor–ligand interaction, thereby modulating the biological functions of these immune cells. Collectively, BayeDEM emerged as a promising tool for oncologists to identify pivotal genes governing mitophagy, thereby enabling mitophagy‐centric therapeutic strategies to counteract drug resistance.

## 1 INTRODUCTION

Mitophagy, a selective cellular process for the degradation of dysfunctional mitochondria, emerged as crucial for maintaining the quality of the mitochondrial pool.^1^ This mechanism holds a dichotomous role in the oncogenesis and progression of cancer, directly participating in the metabolic reprogramming of cancer cells.^2^ It influences tumorigenesis and progression through the regulation of oxidative stress, cellular differentiation, stemness and the immune microenvironment.^2^, ^3^ For example, HIF‐1α, by upregulating BNIP3 and NIX, impairs mitochondrial quality, thereby affecting cellular oxygen consumption and the glycolytic process.^4^

Immune escape, a pivotal factor in cancer progression, enables tumour cells to elude detection and destruction by the immune system.^5^ Mitophagy could affect the immune system by regulating the secretion of mitochondrial DNA (mtDNA) and other damage‐associated molecular patterns (DAMPs), which are recognizable by immune cells.^6^ By controlling the emission of these signals, mitophagy could modulate the activation of immune responses against cancer cells.^6^ Cancer cells undergo metabolic alterations that could foster an immunosuppressive microenvironment, facilitating immune escape. Mitophagy underpins metabolic flexibility in cancer cells, including the Warburg effect, potentially contributing to the immunosuppressive conditions that permit cancer cells to circumvent immune surveillance.^7^ Furthermore, mitophagy could bestow resistance upon cancer cells against therapies that induce mitochondrial damage, including those aimed at provoking an immune response against tumours.^8^ This resistance may indirectly facilitate immune escape by enabling cancer cells to thrive and proliferate despite immune‐mediated challenges. However, an efficacious method to identify the key genes involved in mitophagy, elucidate their roles in tumour progression and immune escape, and apply this knowledge to combat cancer drug resistance, remains elusive.

Advancements in deep learning technologies have facilitated the unveiling of the enigmatic interrelations between mitophagy and cancer immunity, metabolism and drug resistance, given their application across a multitude of clinical conundrums. For example, our preceding investigations proposed an enhanced deep learning framework, augmented with self‐attention mechanisms, demonstrating outstanding performance in the prognostication of cancer drug resistance.^9^ Furthermore, deep learning models, such as artificial neural networks, have empowered the prognostication of outcomes in various diseases, including renal clear cell carcinoma.^10^ The identification of pivotal genes regulating mitophagy inherently represents a feature selection challenge. However, the hyperdimensional nature of RNA‐seq data exacerbates the difficulty of feature selection. The Elastic Net, an advanced deep learning algorithm that synergistically integrates L1 and L2 penalties, is particularly suited for feature selection.^11^ Nonetheless, deep learning models are notoriously recognized as “black‐box” models,^9^ precluding the direct quantification of their decision‐making processes and, consequently, the quantitative assessment of each gene's contribution to the mitophagy phenotype. Therefore, it is imperative to specifically develop a deep learning framework capable of accurately and efficiently identifying the key genes regulating mitophagy.

Tuning hyperparameters represents a pivotal phase in crafting machine learning algorithms, aiming to enhance algorithm efficacy through the judicious selection of optimal hyperparameter configurations. The evolution of this procedure has seen the adoption of diverse strategies, each characterized by its unique benefits and limitations. Primitive techniques such as manual exploration permitted analysts to assess various hyperparameters through a hands‐on approach, yet this tactic proved to be laborious and less effective for intricate models. To advance beyond these limitations, methodologies such as grid and random searches were developed.^12^ The grid search method methodically examines a specified array of hyperparameters, although it incurs significant computational demands by assessing every conceivable permutation. Conversely, the random search strategy selects parameter combinations in a stochastic manner, which in certain scenarios, allows for the identification of suitable parameters more swiftly and with fewer evaluations.^12^ The refinement of hyperparameter tuning methodologies has precipitated the emergence of more complex approaches, including Monte Carlo Tree Search (MCTS) and notably, Bayesian optimization. The latter is distinguished by its capacity for efficient traversal through the hyperparameter landscape, leveraging a probabilistic model of the objective function.^13^ This model forecasts the hyperparameters most likely to yield fruitful outcomes, thereby diminishing the requisite number of evaluations to ascertain optimal configurations. Within the realm of Bayesian optimization, the Tree‐structured Parzen Estimator (TPE) stands out as a superior technique. By estimating the likelihood of a given hyperparameter set's success based on historical data, TPE enables a targeted and more streamlined search, offering a stark contrast to conventional strategies such as grid or random search. The transition towards Bayesian optimization, with a specific emphasis on TPE, underscores a notable evolution in hyperparameter tuning. This progression facilitates a more refined and efficient optimization methodology, capable of dynamically pinpointing the most effective configurations for machine learning models.

In this study, we proposed BayeDEM (Bayesian‐optimized Deep learning for identifying Essential genes of Mitophagy), a computational and automated advanced deep learning architecture devised for the identification of key genes regulating mitophagy in human cancer. For complex and high‐dimensional transcriptomic and clinical data, BayeDEM specifically deployed a feature selection module, incorporating TPE, a Bayesian optimizer, and Elastic Net for automated hyperparameter tuning and handling the hyperdimensionality of transcriptomic data. Subsequently, BayeDEM utilized a modelling module combining CatBoost with TPE, and an elucidation module employing the SHAP algorithm to interpret the decision‐making process of BayeDEM and identify critical genes regulating mitophagy. As a result, BayeDEM precisely modelled the relationship between transcriptomic data and mitophagy, identifying several key genes. One such gene, CERS1, was utilized to develop a drug combination strategy against cisplatin resistance, which was validated in an osteosarcoma‐resistant cell model, elucidating its molecular mechanism in combating drug resistance. Finally, the role of mitophagy in cancer progression, immunity and development was elucidated using scRNA‐seq.

## 2 METHODS

### 2.1 Patients

The single‐cell RNA‐seq (scRNA‐seq) data of 11 osteosarcoma patients was received from the published dataset (GSE152048).^14^ Moreover, scRNA‐seq data of six osteosarcoma patients was obtained from gene expression omnibus (GSE162454).^15^ Seurat package (version 4.3) in R was employed to process scRNA‐seq data.

The transcriptomic data and clinical information of 88 osteosarcoma patients were received from the Therapeutically Applicable Research to Generate Effective Treatments (TARGET). Data were collected from 25 September to 3 November 2022. In the present study, the raw count of transcriptomic data was converted to TPM (Transcripts Per Million or Transcripts Per kilobase of exon model per Million mapped reads) using R software (version 4.3.1), as raw counts were not acceptable for any deep learning models.

### 2.2 BayeDEM architecture

BayeDEM taken transcriptomic data and mitophagy phenotype as its input. Specifically, BayeDEM complied with three core modules, i.e., feature selection, modelling and elucidation. The feature selection and modelling modules were optimized by a Bayesian optimizer.

Feature selection module complied with iterative logistic regression,^16^ Bayesian optimizer (Tree‐Structured Parzen Estimator) and elastic net. In deep learning and machine learning, hyperparameters, which were predetermined, serve to govern the learning algorithm's process. In contrast, the values of other parameters were derived through the training phase. Bayesian optimization was an effective method for optimizing costly objective functions, especially beneficial in the hyperparameter tuning of machine learning models. This strategy excelled when evaluations were time‐intensive and computational resources were scarce.^17^ The elastic net was an advanced network that combined L1 and L2 penalty for feature selection.^18^ All algorithms were implemented with Python (version 3.8.12). Specifically, the statsmodels package was used for conducting iterative logistic regression,^19^ scikit‐learn package for elastic net.^20^

Modelling module complied Bayesian optimizer (Tree‐Structured Parzen Estimator) and CatBoost (Categorical Boosting) to fit the relationship between gene expression profiling and mitophagy phenotype. CatBoost was a gradient boosting library that excels at handling categorical features directly, without the need for extensive preprocessing required by other machine learning algorithms.^21^ The deployment of CatBoost was depended on CatBoost package in Python.

Elucidation module deployed SHAP (SHapley Additive exPlanations) algorithm to quantify the impact of all genes upon mitophagy phenotype (model outputs). SHAP, grounded in cooperative game theory, elucidated the output of machine learning models through the concept of Shapley values. This approach sheds light on the contribution of each feature to an individual instance's prediction, offering a unified metric for assessing feature significance. It enabled interpretable explanations of the model's decision‐making process.^22^ The present elucidation module implemented SHAP by using SHAP package in Python.

### 2.3 BayeDEM performance testing

The performance of BayeDEM was cross‐tested in the training and test set. The discrimination was determined by calculating the area under the receiver operating characteristic (ROC) curve (AUC). The precision and recall ability was tested by using AUC of precision‐recall (PR) curve.

### 2.4 Dissecting mitophagy phenotype and its roles within tumour progression and immune escape

#### 2.4.1 Sample‐level analyses

The critical genes positively regulating mitophagy were received from Gene Ontology database, including PRKN, SLC25A4, HIF1A, STUB1, IRGM, CERS1, NOD2, SLC25A5, FBXO7, VDAC1, HTT, AMBRA1 and VPS13D. All of these genes were used to detect mitophagy phenotype within sample level by using bulk RNA‐seq data. The Non‐Negative Matrix Factorization (NMF), an advanced machine learning algorithm was used for detecting such phenotype in R.^23^ Survival analyses were conducted by using survival curve. The independent analyses were implemented by using univariable Cox regression and multi‐variable Cox regression according to our previous study.^10^, ^16^, ^24^ Similarly, the biological function analyses by using GO enrichment, Gene Set Enrichment Analyses (GSEA) were also according to our previous study.^9^ Briefly, the clusterprofiler package was used for enrichment analyses in R. For immune microenvironment analyses, the ESTIMATE package was used for estimating the overall microenvironment and single sample GSEA (ssGSEA) was used to detect each immune cells' infiltration according to the previous study.^9^

#### 2.4.2 Single cell‐level analyses

DoubletFinder (version 2.0.3) was employed to identify and eliminate doublets,^25^ followed by the exclusion of low‐quality cells based on criteria such as RNA counts below 500, gene counts outside the range of 300 to 6000, or mitochondrial gene content exceeding 25%. Subsequently, gene expression normalization and batch effect correction across various samples were conducted using SCTransform and canonical correlation analysis. For Principal Component Analysis (PCA), the top 2000 variable genes were selected, with the resulting Principal Components (PCs) utilized for cell clustering into distinct populations. These clusters were then characterized using established biomarkers to define their biological identity. In the final step, genes that were differentially expressed and those serving as biomarkers for each cell type were determined, requiring a |log2 fold change| greater than 0.25 and an adjusted *p*‐value of less than 0.05.

AUCell algorithm was conducted to estimate the mitophagy phenotype and stemness of each single cell with established gene set. The stemness gene set included ABCG2, BMI1, CD34, CD44 and CTNNB1. EPAS1, EZH2, HIF1A, KDM5B, KLF4, LGR5, MYC, NANOG, NES, NOTCH1, POU5F1, SOX2, TWIST1, ZFP42, ZSCAN4.

Transfer deep learning was utilized to project scRNA‐seq data onto a pre‐learned cell cycle space, following which cell‐cycle pseudo‐time was inferred as the polar angle around the origin, ranging from [0, 2π]. Subsequently, discrete cell cycle stages were deduced using the Schwabe method. All analyses were conducted using the Tricycle (version 1.10.0) R package.^26^

The Monocle3 R package (version 1.3.1) was used to deduce cellular developmental trajectories.^27^ By employing dimensionality reduction and clustering techniques, Monocle3 identifies changes in gene expression across cells relative to their advancement along a developmental pathway. This is achieved by constructing a principal graph in the reduced dimensionality space with a reverse graph approach. Cells are then arranged in a sequence referred to as ‘pseudotime’, illustrating their sequential positioning on the developmental trajectory. To identify genes whose expression varies along this trajectory, Moran's *I* test was applied. Furthermore, gene expression patterns were grouped into co‐expression modules using Leiden clustering in combination with the K‐Nearest Neighbours algorithm, facilitating the analysis of gene co‐regulation across cellular development.

By employing CellChat (version 1.6.1), which combines elements of social network analysis, pattern recognition and various learning techniques, we were able to quantitatively analyse and contrast inferred intercellular communication networks.^28^, ^29^ This approach uses an extensive receptor–ligand database to deduce interactions among different cell types. CellChat determines the key players in cell‐to‐cell communication—such as the primary senders, receivers, mediators and influencers—using metrics derived from a weighted directed network. These metrics include out‐degree, in‐degree, flow betweenness and information centrality. Moreover, CellChat utilizes non‐negative matrix factorization to reveal important signals and uncover latent patterns of communication across signalling pathways.

### 2.5 Experimental analyses

#### 2.5.1 Cell culture

The human osteosarcoma cell line, 143B, was purchased from Procell (China). The 143B cells were maintained in Minimum Essential Medium (Gibco, USA) with 10% fetal bovine serum (Gibco) and 1% Penicillin–Streptomycin (P–S) solution (Gibco). For establishing cisplatin (CDDP)‐resistant 143B cells (143B/CDDP), the present study treated cells with increasing doses of CDDP. The compound identified as P053, characterized by its chemical configuration as (S)‐2‐amino‐4‐(4‐(3,4‐dichlorobenzyloxy)phenyl)‐2‐methylbutan‐1‐ol, exhibits the capacity to lower the peak rate of reaction while having minimal impact on the affinity for the substrate, thus suggesting its role as a non‐competitive inhibitor. This substance significantly obstructs CerS1 activity, as evidenced by an IC50 value of 0.5 μM, underscoring its efficacy and selectivity.^30^ For the treatment of 143B/CDDP cells, P053, the CerS1 inhibitor, was purchased from medchemexpress (MCE, USA) and CDDP was obtained from Yuanye Bio‐Technology (China). 143B/CDDP cells were treated with 5 μM CDDP and 1.5 μM P053.

#### 2.5.2 Cell viability, proliferation, migration, invasion and colonies formation measurement

After treating 143B/CDDP cells for 2 days, the present study measured its cell viability, proliferation, migration, invasion and colony formation under different treatments. The cell viability was used CCK‐8 Kit according to the instruction of provider (Beyotime, China), proliferation was used EdU assay to detect. The transwell assay was employed to test the invasion, the cell wound healing assay and the colonies formation assay were used to test migration and colony formation of 143B/CDDP cells according to our previous study.^31^

#### 2.5.3 Flow cytometry, terminal deoxynucleotidyl transferase (TdT) dUTP Nick‐End Labeling (TUNEL) assay, mitochondrial membrane potential and mitochondrial dynamics

Apoptosis levels were quantified via flow cytometry, employing 7‐AAD and PE‐conjugated Annexin V for cell labelling, as per the guidelines provided by the kit's manufacturer (Yeason, Shanghai, China). To detect nuclear DNA fragmentation signalling late‐stage apoptosis in cells and tissues, the TUNEL Apoptosis Detection Kit (Yeason) was applied. The assessment of mitochondrial membrane potential was performed utilizing the JC‐1 Enhanced Mitochondrial Membrane Potential Assay Kit (Beyotime, Shanghai, China). MitoTracker Red staining was executed in alignment with methods delineated in earlier research.^32^

### 2.6 Statistical analysis

The statistical analyses were conducted with R software (version 4.3.1). The Student's *t*‐test was used to compare data between two groups, and one‐way ANOVA and Tukey's tests were used to compare data among more than two groups. A *p*‐value of <0.05 was considered significant.

## 3 RESULTS

### 3.1 BayeDEM methodology

**FIGURE 1:**
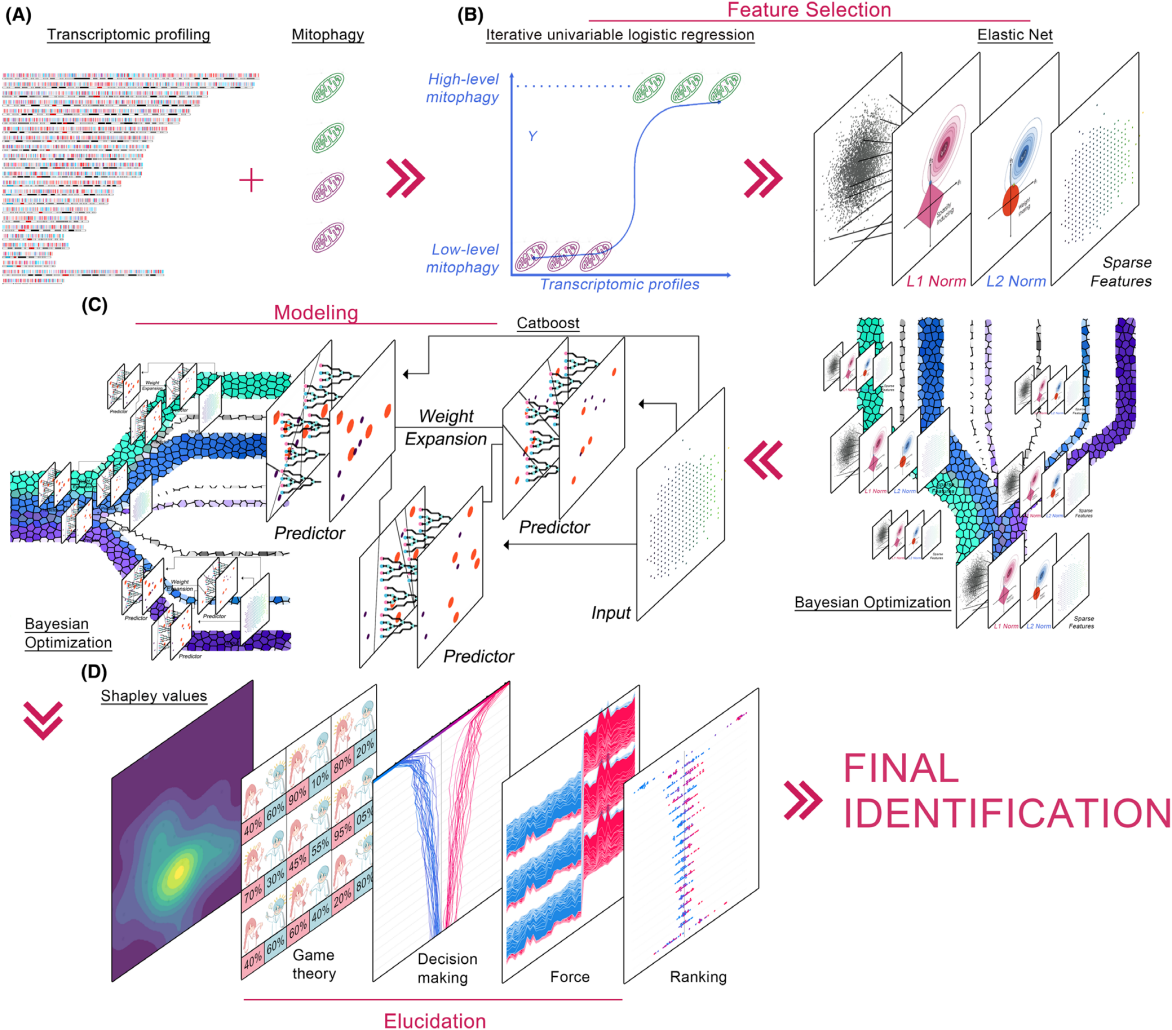
BayeDEM architecture. (A) Inputted data. (B) Feature selection module. (C) Modelling module. (D) Elucidation module.

The Bayesian‐optimized Deep learning for identifying Essential genes of Mitophagy (BayeDEM) was developed as a sophisticated, automated deep learning architecture designed for the precise identification of pivotal genes governing mitophagy in human cancers. BayeDEM received transcriptomic profiles and mitophagy phenotypes as inputs (Figure 1A). Given the exceptionally high‐dimensional nature of transcriptomic data, direct modelling of the input data was inherently susceptible to the “curse of dimensionality.” The “curse of dimensionality” refers to the challenges and inefficiencies that arise when dealing with high‐dimensional data, including data sparsity, diminished utility of distance metrics, increased risk of overfitting and computational complexity. These phenomena make data analysis and predictive modelling more difficult as the number of dimensions (features) increases. Additionally, the decision‐making process of deep learning models was characteristically non‐transparent, rendering the pinpointing of essential genes a complex endeavour. To address these challenges, BayeDEM compiled three distinct modules: feature selection, modelling and elucidation (Figure 1).

The feature selection module was tasked with processing input data to discern features intimately associated with outcomes, thereby reducing dimensionality (Figure 1B). Initial stages involved the application of iterative univariable logistic regression to preliminarily identify salient features impacting the mitophagy phenotype. This was followed by the deployment of the Elastic Net, a sophisticated regularization network that elegantly combined the L1 (least absolute shrinkage and selection operator) and L2 penalties (*Tikhonov* regularization), aimed at excluding features exhibiting collinearity or negligible influence on the mitophagy phenotype. The L1 regularization promotes sparseness within model coefficients, possibly nullifying certain coefficients, thereby conducting feature selection. Conversely, L2 regularization seeks to uniformly reduce the magnitude of all coefficients without driving them to zero, resulting in models that contemplate all features, albeit with moderated influence. Specifically, while the L1 regularization's penalty function was articulated as ${\left|\left|\beta \right|\right|}_{1}=\sum_{j=1}^{p}\left|\beta_{j}\right|$, the Elastic Net introduced a quadratic component ${\left|\left|\beta \right|\right|}^{2}$, formulating its estimates as $\hat{\beta }\equiv \text{argmin}\left({\left|\left|y-\mathrm{X\beta}\right|\right|}^{2}+\lambda_{2}{\left|\left|\beta \right|\right|}^{2}+\lambda_{1}{\left|\left|\beta \right|\right|}_{1}\right)$. Such modifications endowed the loss function with strong convexity, ensuring a unique minimum.

Despite the potential of deep learning models, their performance was contingent upon the optimal combination of hyperparameters. Suboptimal hyperparameters frequently resulted in inferior model fitting and outputs. Consequently, BayeDEM employed the Tree‐Structured Parzen Estimator (TPE) as a Bayesian optimizer for hyperparameter tuning (Figure 1B,C). By integrating prior knowledge regarding the objective function and refining this knowledge based on the outcomes of objective function evaluations, Bayesian optimization endeavoured to identify the global optimum with the minimum number of evaluations feasible. Unlike alternative surrogate models, such as Gaussian Processes, TPE elucidated the objective function by establishing a probabilistic model for the likelihood $p\left(x|y\right)$ of observing a hyperparameter configuration *x* given its associated objective value *y*, alongside a prior $\;p\left(x\right)$ over the hyperparameter space. Mathematically, the TPE algorithm articulated $p\left(x|y\right)$ by fitting two distinct Parzen window density estimators to the hyperparameters in the regions delineated as good and bad, respectively. TPE selected the optimal hyperparameter combination by maximizing the Expected Improvement (EI) criterion, which for BayeDEM's minimization problem could be formulated as follows: $\mathrm{EI}\left(x\right)=\int_{-\infty }^{y^{*}}\left(y^{*}-y\right)p\left(y|x\right)\mathrm{dy}$. Where, $y^{*}$ was the best objective value observed so far. Within BayeDEM architecture, Bayesian optimizer was employed to optimize Elastic Net in feature selection module and CatBoost (Categorical Boosting) in Modelling module (Figure 1B,C).

The modelling module deployed the TPE algorithm alongside CatBoost (Figure 1C). CatBoost, distinguished as a gradient boosting architecture, epitomized an ensemble learning stratagem that meticulously constructed its model through a sequential approach. It consecutively incorporated decision trees, each subsequent arboreal structure ameliorating discrepancies occasioned by its antecedents. The training data was denoted as $D={\left\{\left(x_{i},y_{i}\right)\right\}}_{i=1}^{N}$, wherein $x_{i}$ delineated the attributes of the i‐th sample, and $y_{i}$ corresponded to the associated mitophagy phenotype. CatBoost's objective was to learn a function $F\left(X\right)$ that accurately emulated the genuine interrelation between $x_{i}$ and $y_{i}$: $F\left(X\right)=\sum_{t=1}^{T}f_{t}\left(x\right)$, wherein $f_{t}\left(x\right)$ was the t‐th decision tree. The loss function of CatBoost could be mathematically summarized as: $F_{t+1}\left(x\right)=F_{t}\left(x\right)+\eta \cdot f_{t+1}\left(x\right)$, where η was learning rate.

The elucidation module employed SHAP (Shapley Additive exPlanations) as a sophisticated deep learning explainer to identify the pivotal genes affecting mitophagy phenotypes (Figure 1D). It furnished profound insights into the contribution of each feature towards the prediction for a singular instance, predicated on the Shapley values concept derived from cooperative game theory. Within the elucidation module of BayeDEM, every genes of a sample were regarded as a “participant” in the game, wherein the “reward” constituted the prediction rendered by the BayeDEM. For a given sample with genes $x=\left\{x_{1},\cdots ,x_{M}\right\}$ and well‐trained BayeDEM f, the SHAP value of j‐th gene was defined as: $\emptyset_{j}=\sum_{S\in \left\{x_{1},\cdots ,x_{M}\right\}\backslash \left\{x_{j}\right\}}\frac{\left|S\right|!\left(M-\left|S\right|-1\right)!}{M!}\left(f_{x}\left(S\cup \left\{x_{j}\right\}\right)-f_{x}\left(S\right)\right)$. Wherein, *S* represented a subset of genes excluding $x_{j}$, and $\left|S\right|$ was the size of S; $f_{x}\left(S\right)$ was the prediction of BayeDEM when only genes belonging to S were used; the term $\frac{\left|S\right|!\left(M-\left|S\right|-1\right)!}{M!}$ deduced the weight for each subset, accounting for all possible orders in which genes could appear; the difference $f_{x}\left(S\cup \left\{x_{j}\right\}\right)-f_{x}\left(S\right)$ estimated the marginal contribution of adding gene $x_{j}$ to subset S. The important gene always earned the larger $\left|\text{SHAP value}\right|$ as compared to others.

### 3.2 Elucidation of mitophagy phenotype and its implications within osteosarcoma

#### 3.2.1 Osteosarcoma's mitophagy phenotype detection via Non‐negative Matrix Factorization (NMF)

**FIGURE 2:**
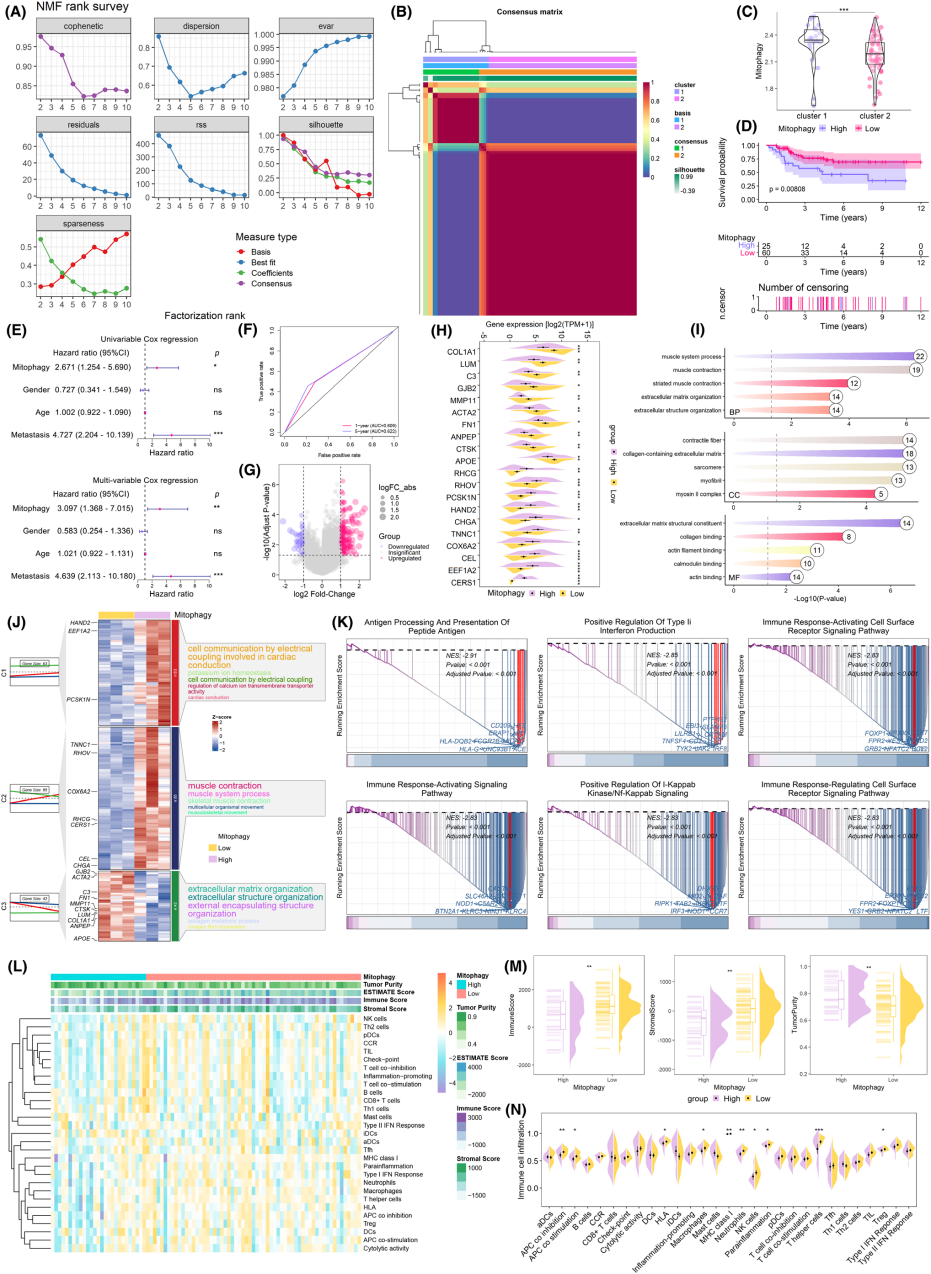
Elucidation of mitophagy phenotype and its biological involvement in osteosarcoma. (A, B) NMF optimization and classification of osteosarcoma's mitophagy phenotype. (C) Mitophagy level in different clusters identified by NMF. (D) Survival analyses. (E) Independent analyses of mitophagy phenotype. (F) ROC curve of mitophagy phenotype. (G, H) Differential expressed genes analyses between high and low mitophagy osteosarcoma. (I, J) GO enrichment of differential expressed genes. (K) GSEA results. (L–N) Immune microenvironment analyses.

The present study employed osteosarcoma as a paradigm to validate the efficacy and utility of BayeDEM. Prior to leveraging BayeDEM for the identification of hub genes regulating osteosarcoma mitophagy, it was important to detect the mitophagy phenotype of osteosarcoma and to explicate the biological and clinical implications of mitophagy in osteosarcoma's progression. Employing 13 critical genes involving mitophagy, NMF, an advanced machine learning technique, was harnessed to detect mitophagy phenotypes within osteosarcoma. The Brunet algorithm was applied to ascertain the optimal hyperparameter rank for NMF, established at 2 (Figure 2A), thus enabling the unsupervised classification of osteosarcoma into two distinct clusters (Figure 2B). Quantification of mitophagy in each osteosarcoma specimen was achieved through ssGSEA, revealing that Cluster 1 manifested a significantly elevated level of mitophagy, henceforth termed the mitophagy high group, whereas Cluster 2 was denoted as the low group (Figure 2C). Prognostic evaluations affirmed that individuals within the high mitophagy group exhibited markedly diminished overall survival (Figure 2D). Mitophagy also emerged as an independent hazardous factor (Figure 2E), which could proficiently prognosticate patient survival (Figure 2F). These findings indicated that mitophagy's enhancement precipitated an unfavourable outcome in osteosarcoma patients, possibly via the augmentation of mitochondrial quality control through increased mitophagy, which in turn facilitates tumour cell endurance against external adversities, including chemotherapeutic interventions and oxidative stress.

#### 3.2.2 High mitophagy contributed to immunosuppression and cellular adhesion of osteosarcoma

Subsequently, the present study investigated the molecular mechanisms underlying the prognostically unfavourable consequences of elevated mitophagy. As resultant data detailed in Figure 2G,H, high‐ and low‐mitophagy osteosarcoma had significant different gene expression patterns, with the differentially expressed genes predominantly regulating cellular communication, muscle development and cell adhesion (Figure 2I,J and Table S1). Further GSEA analyses suggested that antigen presentation, interferon responses and various immune responses were dysregulated in high‐mitophagy osteosarcoma, indicating a pivotal role of immunity in its progression (Figure 2K). Therefore, the tumour microenvironment of osteosarcomas with differing levels of mitophagy was further elucidated. As illustrated in Figure 2L,M, osteosarcomas with high mitophagy exhibited higher tumour purity and lower levels of overall immune cell infiltration, confirming an immunosuppressive microenvironment. Specifically, the infiltration levels of various tumour‐killing cells, including dendritic cells, neutrophils and macrophages, were significantly reduced in high‐mitophagy osteosarcoma (Figure 2N), suggesting that excessively active mitophagy contributed to the immune evasion of osteosarcoma cells.

### 3.3 BayeDEM performed outstanding performance in identifying osteosarcoma mitophagy

**FIGURE 3:**
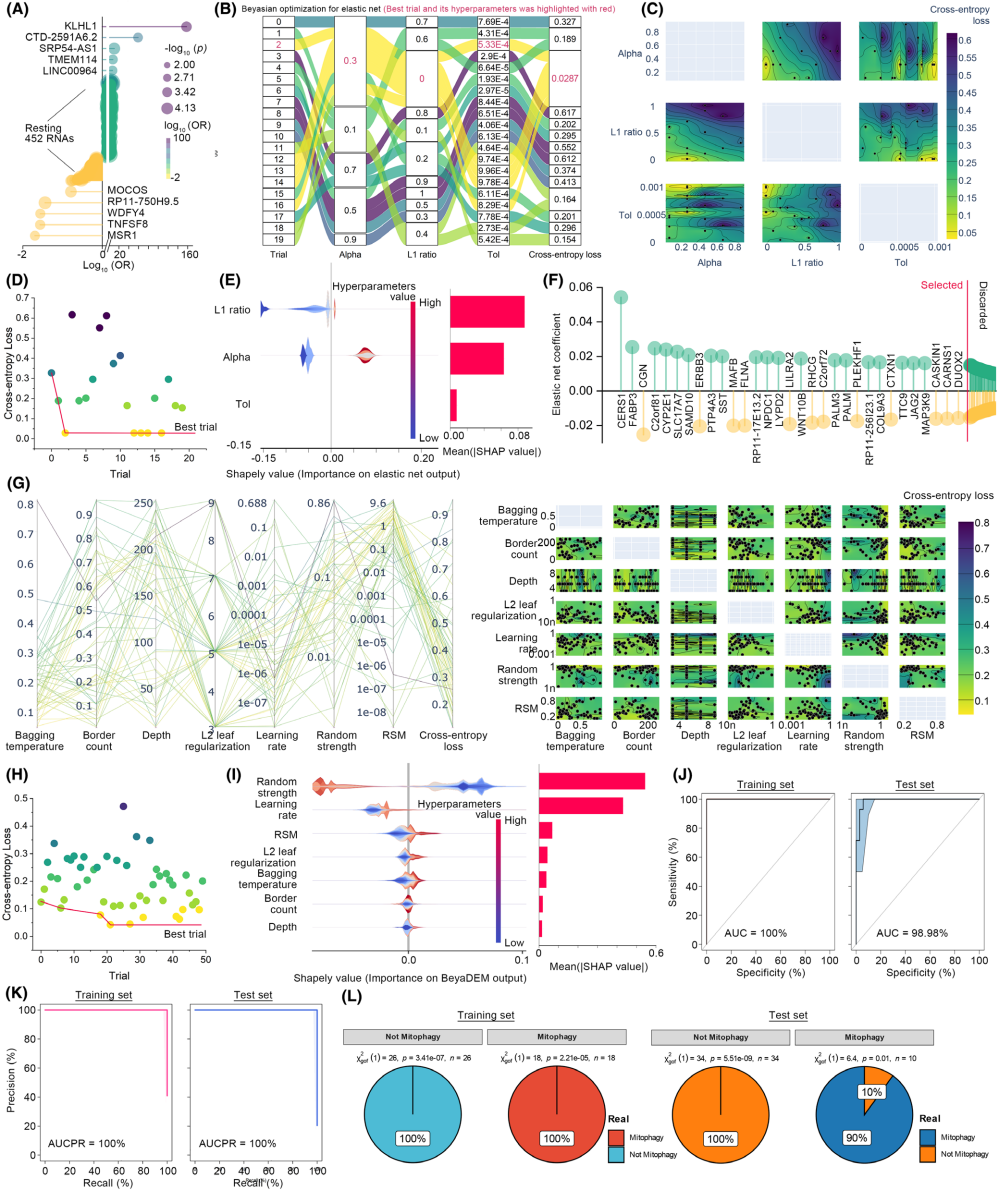
Hyperparameter tuning, modelling and performance test of BayeDEM. (A) Univariable logistic regression results. (B, C) Bayesian optimization of Elastic Net. (D) Optimization history of Elastic Net. (E) Hyperparameters' importance of Elastic Net. (F) Elastic Net identified features. (G) The Bayesian optimization of hyperparameters of BayeDEM's modelling module. (H) Hyperparameters tuning history of BayeDEM's modelling module. (I) Hyperparameters' importance of BayeDEM's modelling module. (J–L) Performance testing of BayeDEM.

All osteosarcoma patients were randomly allocated into training and test sets, each constituting 50% of the total sample. Subsequently, BayeDEM was employed to train a predictive model utilizing solely the training set, which was then independently tested within the test set. Initially, the transcriptomic data and mitophagy phenotypes of osteosarcoma specimens were fed into BayeDEM's feature selection module. Through iterative univariable logistic regression, the first phase of this module identified 462 hub genes that exerted a considerable influence on the mitophagy phenotype (*p* < 0.001). The top five hazardous and protective genes were elucidated in Figure 3A. Thereafter, these genes were subjected to a Bayesian‐optimized elastic net, where the TPE optimizer conducted 20 trials to ascertain the optimal hyperparameter configuration, utilizing cross‐entropy loss as the criterion for evaluation (Figure 3B,C). The tuning process for hyperparameters culminated in the second iteration, identifying the most favourable combination as Alpha 0.3, L1 ratio 0 and Tolerance 5.33E‐4, with a cross‐entropy loss recorded at 0.0286710 (Figure 3D). Notably, the L1 ratio emerged as the paramount contributor to the model's performance, as underscored by the highest mean absolute SHAP value among all considered hyperparameters (Figure 3E). Conclusively, the feature selection procedure isolated 31 essential genes for further modelling (Figure 3F).

The BayeDEM modelling module accepted the refined outcomes from its feature selection module. Within this module, the TPE optimizer initiated 50 trials to identify the ideal hyperparameter combination across the defined parameter space (Figure 3G), achieving this objective on the 21st trial (Figure 3H). The supreme hyperparameter configuration comprised a Depth of 5, L2 leaf regularization of 2.846E‐07, Bagging temperature of 0.5876156188875777, Border count of 206, Learning rate of 0.06717152873551722, Random strength of 4.448312976713604 and RSM of 0.26452660853106685, culminating in a minimum cross‐entropy loss of 0.04241866685354354. Predominantly, Random strength emerged as the critical determinant of BayeDEM's performance (Figure 3I), succeeded by the Learning rate, RSM, etc. Subsequent cross‐validation of BayeDEM's performance across both training and test sets substantiated its exceptional discriminative capacity, enabling the precise differentiation of varied mitophagy phenotypes with a remarkable AUC of 98.96% in the test set (Figure 3J). Furthermore, BayeDEM demonstrated flawless precision, as depicted by an area under the precision‐recall curve of 100% within both train and test sets (Figure 3K). Moreover, BayeDEM demonstrated the outstanding consistency between its predictions and actual observations (Figure 3L). Collectively, BayeDEM excellently fitted the relationship between RNA expression patterns and mitophagy in osteosarcoma patients.

### 3.4 BayeDEM highlighted CERS1 as the hub gene regulating mitophagy of osteosarcoma

**FIGURE 4:**
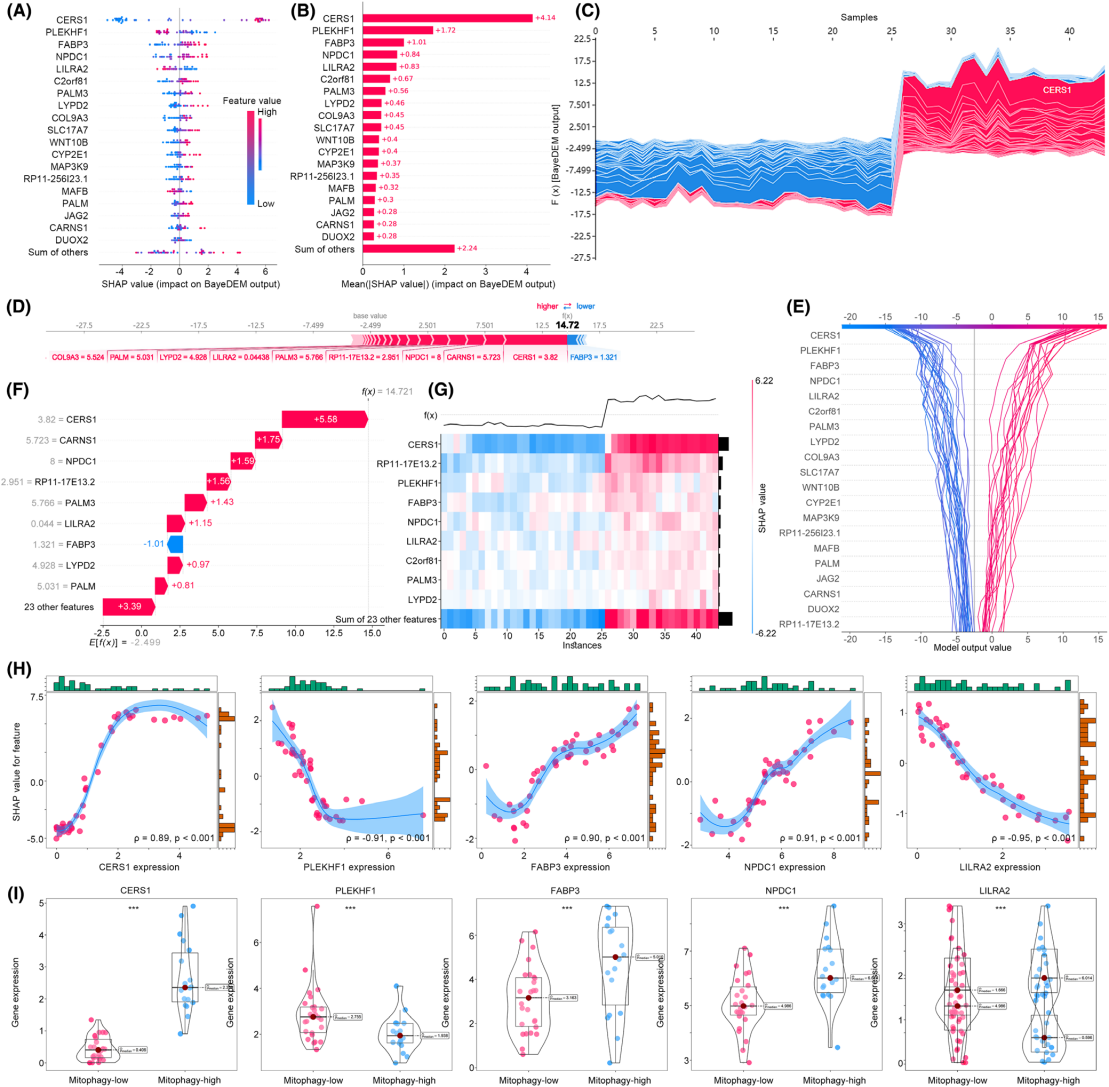
Identification of hub genes regulating osteosarcoma mitophagy via BayeDEM's elucidation module. (A, B) SHAP summary plot. (C) SHAP force plot. (D, F) The decision‐making of BayeDEM upon one osteosarcoma patient. (G, E) the overall decision‐making process of BayeDEM upon all samples. (H) SHAP dependency plots. (I) The expression of identified hub genes between different mitophagy phenotypes.

SHAP was employed to interpret the decision‐making process of BayeDEM, focusing on identifying pivotal genes influencing the osteosarcoma mitophagy. Initially, the SHAP algorithm calculated the SHAP values for all genes, followed by their ranking to pinpoint critical genes. The top 20 hub genes identified by SHAP were detailed in Figure 4A,B, with CERS1 emerging as the most influential (mean(|SHAP value|): 4.14), followed by PLEKHF1 (1.72), FABP3 (1.01), NPDC1 (0.84), LILRA2 (0.83), etc. Specifically, SHAP illuminated the manner in which BayeDEM processed the expression levels of each gene, aggregating them into the final model output (Figure 4C and Figure S1). Taking an osteosarcoma sample as an illustration, CERS1's expression exerted the most substantial positive influence on the sample's BayeDEM prediction value of 5.58, propelling the model prediction towards a positive label indicative of high mitophagy (Figure 4D,F). Concurrently, CARNS1 and FABP3 also contributed positively or negatively to the model's ultimate output based on their expression levels in the sample. The contribution of SHAP‐identified hub genes to BayeDEM's prediction values was highlighted in Figure 4G, while the decision‐making process involving these key genes was depicted in Figure 4E. Subsequently, the SHAP dependency of the top five genes was calculated to further elucidate their impact on model predictions, specifically their capability to drive predictions towards high or low mitophagy (Figure 4H). CERS1, FABP3 and NPDC1 significantly propelled predictions towards high mitophagy, indicating these genes positively regulate mitophagy in osteosarcoma. Conversely, higher expressions of PLEKHF1 and LILRA2 led to predictions of low mitophagy. Gene expression comparisons between groups corroborated these findings, demonstrating significantly higher expressions of CERS1, FABP3 and NPDC1 in high‐mitophagy osteosarcoma, whereas PLEKHF1 and LILRA2 were significantly less expressed (Figure 4I).

### 3.5 Inhibition of CERS1‐sensitized CDDP‐resistant osteosarcoma cells to CDDP by disrupting the mitophagy and mitochondrial quality control

**FIGURE 5:**
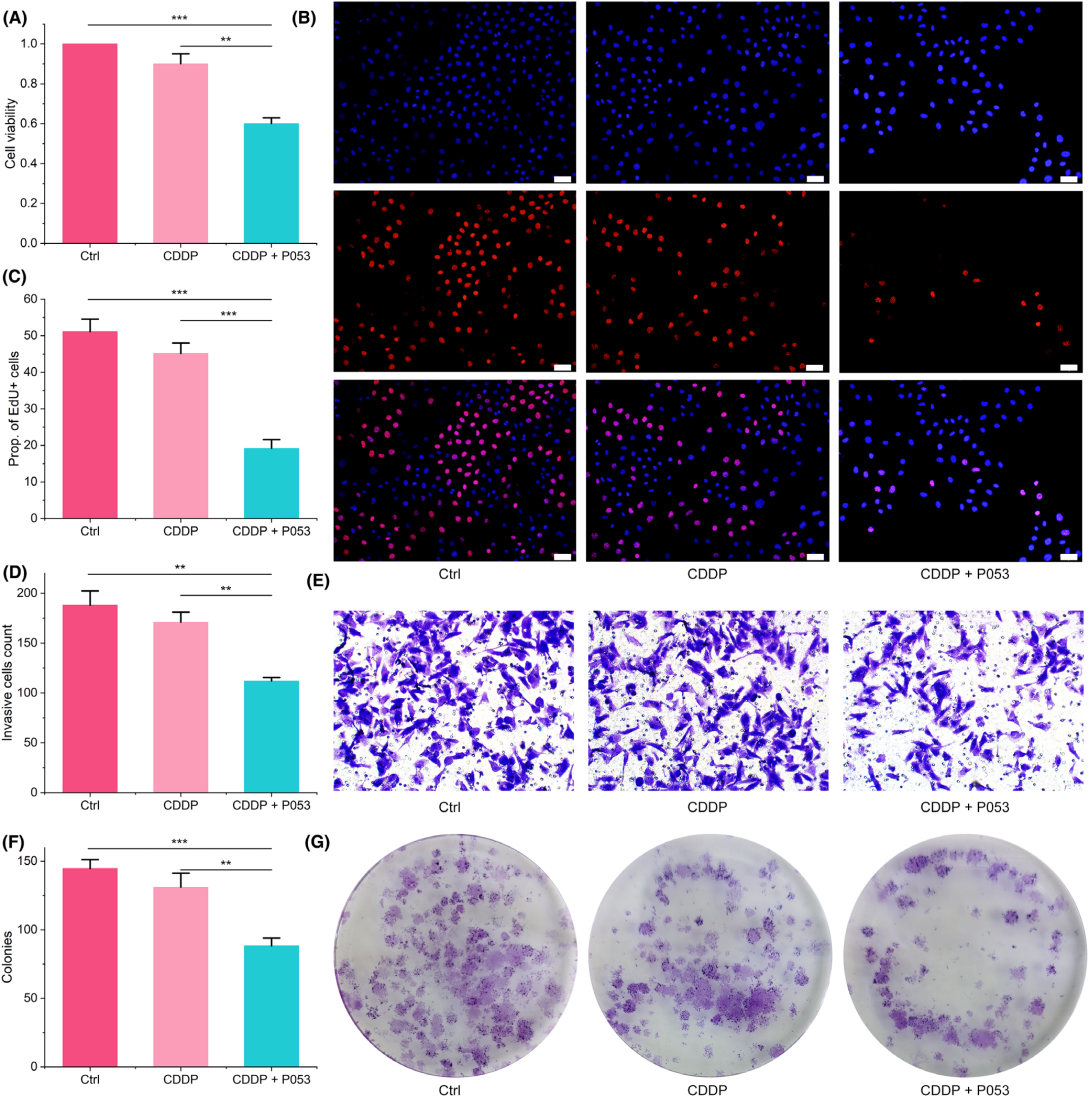
The effect of CERS1 inhibition upon malignant behaviour of CDDP‐resistant osteosarcoma cells. (A) CCK‐8 results. (B, C) EdU assay to measure cell proliferation. (D, E) transwell assay to test cell invasion. (F, G) Cell colony formation.

**FIGURE 6:**
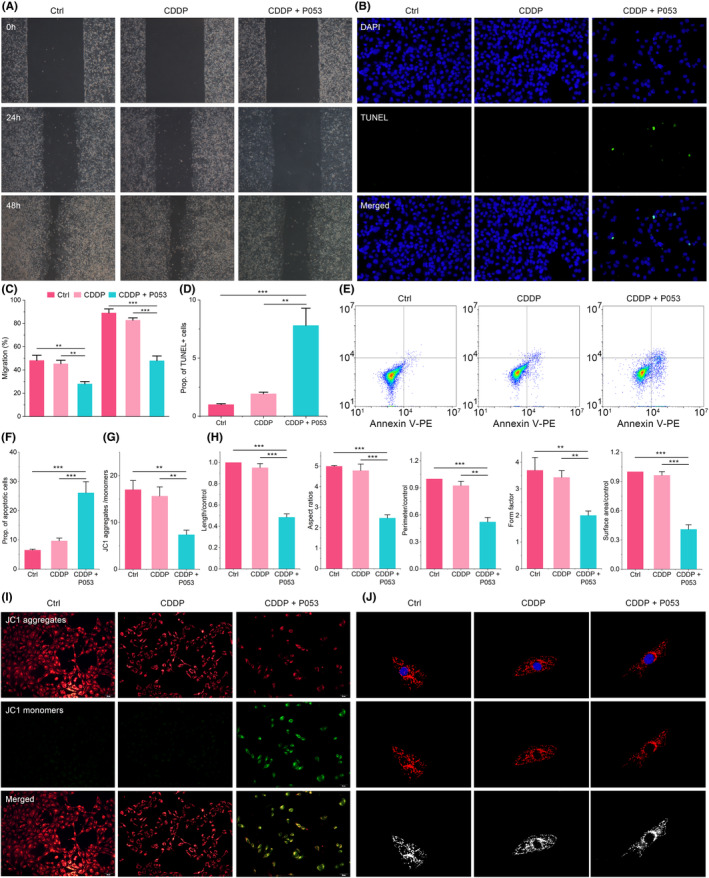
The effect of CERS1 inhibition upon malignant behaviour and mitochondria of CDDP‐resistant osteosarcoma cells. (A, C) Cell wound healing assay to test migration, (B, D) TUNEL assay to measure cell apoptosis. (E, F) Overall apoptosis rate measurement. (G, I) The potential of mitochondrial membrane measurement. (H, J) Mitochondrial dynamics.

BayeDEM identified CERS1 as the paramount gene positively regulating mitophagy in osteosarcoma. Clinical treatment protocols for osteosarcoma, such as neoadjuvant chemotherapy, are principally designed to induce apoptosis in osteosarcoma cells. For example, CDDP promotes apoptosis in osteosarcoma cells via the mitochondrial pathway by augmenting oxidative stress. However, osteosarcomas typically utilize a variety of mechanisms to rapidly remove mitochondria that are of poor quality. One such mechanism is mitophagy, which plays a crucial role in preventing the onset of mitochondrial‐dependent apoptotic pathways. This prevention is achieved by ensuring the removal of damaged mitochondria before they can release cytochrome C (Cyt C). The release of Cyt C from these compromised mitochondria is a key trigger for initiating the mitochondrial‐dependent apoptotic pathways. Therefore, by efficiently eliminating damaged mitochondria through mitophagy, osteosarcomas can effectively circumvent the initiation of these apoptotic processes. Consequently, inhibiting mitophagy by suppressing CERS1 disrupts the mitochondrial quality control mechanisms of osteosarcoma cells, thereby countering their CDDP resistance. The present study utilized small molecule inhibitor P053 of CERS1 to downregulate the expression of CERS1 in the CDDP‐resistant osteosarcoma cell line (143B/CDDP). The results, as depicted in Figure 5A, demonstrate that CDDP alone had a minimal impact on cellular viability, whereas the combination with P053 resulted in a significant reduction in cell viability. The EdU assay further corroborated that the combined treatment with CDDP and P053 significantly inhibited the proliferative potential of 143B/CDDP cells (Figure 5B,C). Additionally, the sensitizing effect of P053 to CDDP treatment also inhibited the prototypical malignant behaviours of the tumour, including invasion (Figure 5D,E), colony formation (Figure 5F,G) and migration (Figure 6A,C).

Mechanistically, P053 disrupted the mitophagy and mitochondrial quality control mechanisms of 143B/CDDP by inhibiting CERS1, thereby sensitizing 143B/CDDP cells to CDDP‐induced apoptosis. Following treatment with P053 and CDDP, 143B/CDDP cells exhibited significant apoptosis, characterized by notable DNA fragmentation (Figure 6B,D), and an increase in the ratio of late apoptotic cells to the total apoptotic cell population (Figure 6E,F). The treatment with P053 led to a reduction in mitophagy in 143B/CDDP, resulting in the inability to timely clear damaged mitochondria, thereby triggering the apoptotic cascade. This was evidenced by the detection of mitochondrial membrane potential and mitochondrial morphology, where treatment with P053 and CDDP led to a significant loss in mitochondrial membrane potential, indicating a breach in the mitochondrial barrier (Figure 6G,I). Similarly, treatment with P053 and CDDP severely impaired the morphology and dynamics of the mitochondria in 143B/CDDP cells, as evidenced by the unfavourable length, aspect ratio, surface area, perimeter and form factor of their mitochondria (Figure 6H,J). In contrast, treatment with CDDP alone was insufficient to disrupt the mitochondrial quality control mechanism.

### 3.6 Quality control, pre‐processing and cell annotation of scRNA‐seq data

Initially, quality control protocols were applied to the scRNA‐seq data from osteosarcoma patients, involving the removal of double‐lets and low‐quality cells. Following this, the analysis identified the top 2000 variable genes, enabling the use of the Canonical Correlation Analysis (CCA) algorithm. The CCA was pivotal for merging scRNA‐seq data across different samples and mitigating batch effects. A subsequent step involved performing PCA to identify the top 15 principal components for cell clustering at a 1.5 resolution. Finally, various biomarkers were utilized to determine the identities of the unique cell clusters: B cells (CD79A, CD19 and MS4A1), CD8+ T cells (CD3D, CD8A and CD8B), CD4+ T cells (CD4, IL7R and CCR7), regulatory T cells (Tregs) (FOXP3 and TNFRSF4), dendritic cell (DCs) (HLA‐DQA1, HLA‐DRB1, CD1C and FCER1A), macrophages (CD163 and CD68), monocyte (CD14, FCGR3A, S100A8 and S100A9), natural killer (NK) cells (CD160, NKG7, GNLY, CD247, CCL3 and GZMB), osteoblasts (COL1A1, CDH11 and RUNX2), chondrocytes (ACAN, COL2A1 and SOX9), osteoclastic (CTSK, MMP9 and ACP5), mesenchymal stem cells (MSCs) (CXCL12, MME, THY1 and SFRP2), endothelial cells (VWF, PECAM1, ACKR1, SELE and CLDN5) and fibroblasts (ACTA2, DCN and LUM).

**FIGURE 7:**
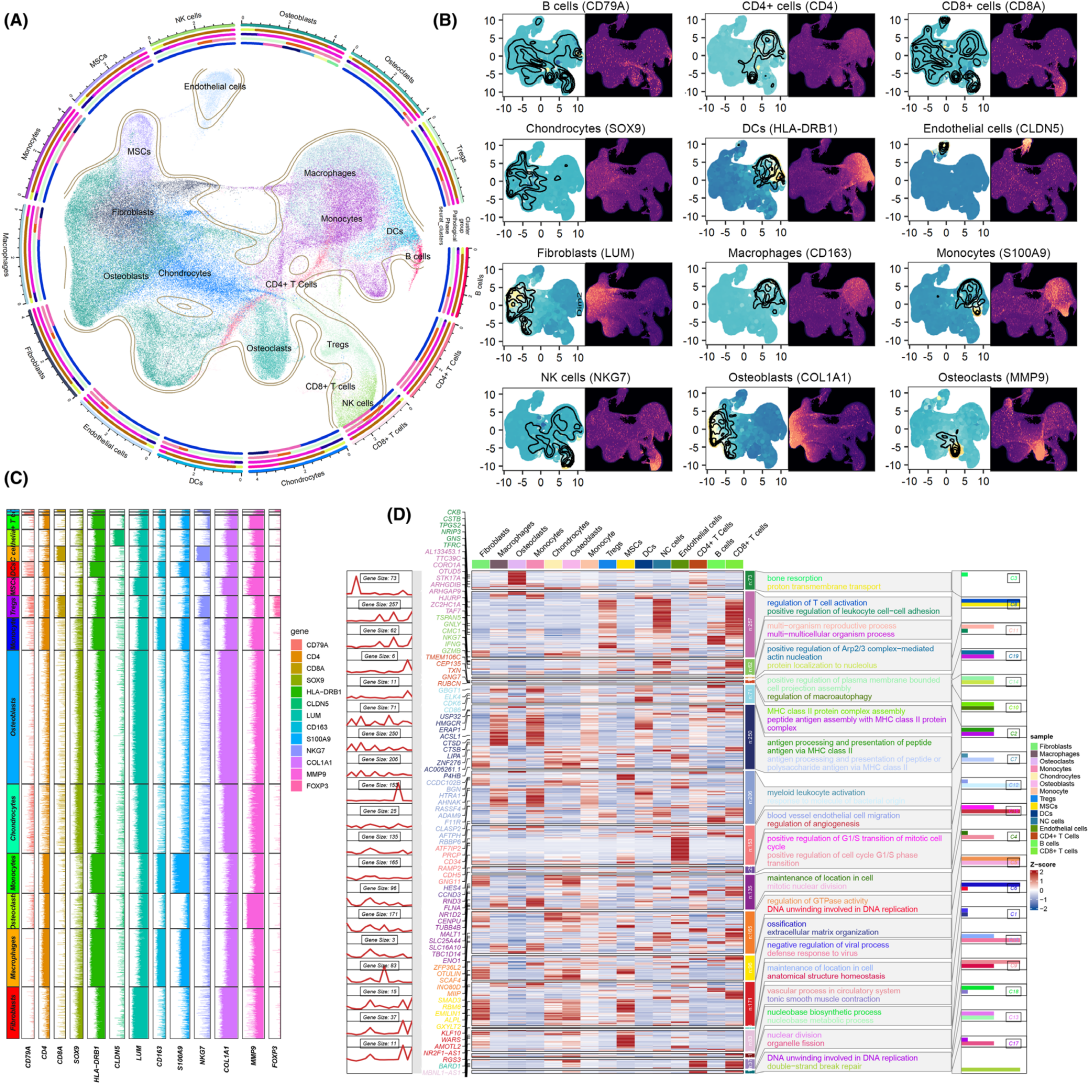
Quality control, pre‐processing and cell annotation of scRNA‐seq data. (A) Clustering and annotation. (B, C) Representative biomarkers to annotate cells. (D) Biological function analyses of each cell type.

The annotated cells were detailed in Figure 7A, and the representative biomarkers to identify each cell were displayed in Figure 7B,C. Afterward, the positively expressed marker gene of every cell cluster was identified by using differential gene expression analyses (adjusted‐*p* < 0.05 and log2 fold‐change > 0.25). These marker genes were fed to GO enrichment to identify the major biological functions of each cell. As results shown in Figure 7D, malignant osteoblasts upregulated many biological processes ranging from proliferation and tumour immunity, including antigen presentation, DNA duplication, mitotic nuclear division, etc., highlighting the active immune involvement of malignant osteoblasts.

### 3.7 Mitophagy mediated malignant osteoblasts' interaction with immune cells, and engaged them in proliferation and tissue‐residency

**FIGURE 8:**
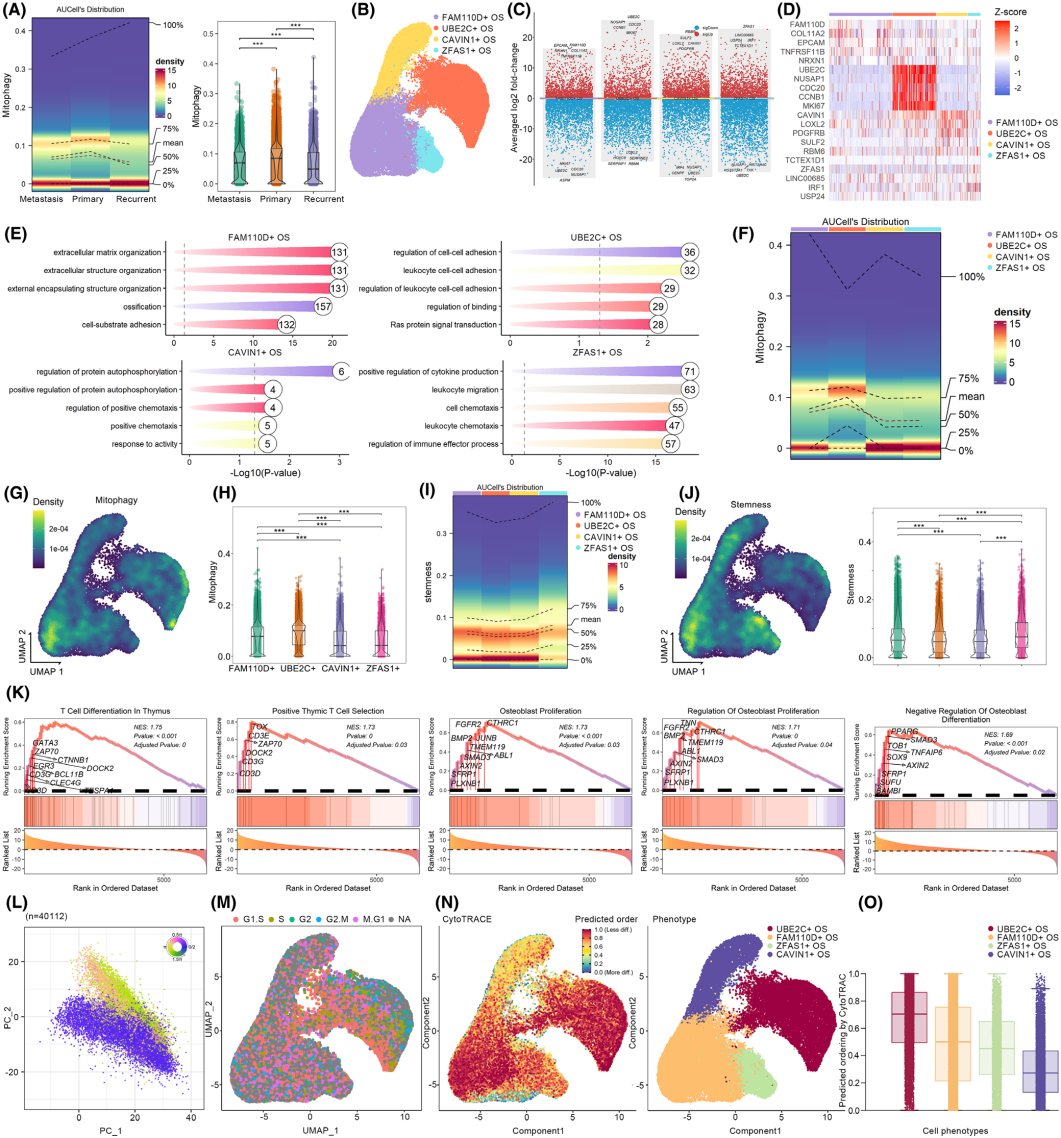
Analyses of mitophagy phenotype and malignant osteoblasts. (A) Mitophagy phenotypes of malignant osteoblasts in different clinical osteosarcoma. (B) Identification of subtypes of malignant osteoblasts. (C, D) Marker gene identification of subtypes of malignant osteoblasts. (E) Biological function of malignant osteoblast subtypes. (F–H) Mitophagy phenotypes analyses of malignant osteoblast subtypes. (I, J) Stemness analyses of malignant osteoblast subtypes. (K) GSEA analyses of malignant osteoblast subtypes. (L, M) Cell cycle analyses of malignant osteoblast subtypes. (N, O) Differentiation potential analyses of malignant osteoblast subtypes.

Osteoblastic osteosarcoma, being of paramount significance within the clinical spectrum of osteosarcomas, was thus the focal point of this investigation, concentrating on malignant osteoblasts. The AUCell algorithm was employed to quantitatively assess the mitophagy phenotype in each malignant osteoblast. As results detailed in Figure 8A, a variable upregulation of mitophagy was observed across malignant osteoblastic cells, most notably in primary osteosarcoma, indicating that mitophagy predominantly influenced the primary osteosarcoma and the tissue‐resident malignant osteoblasts. Consequently, the present study further clustered malignant osteoblasts into four distinct subgroups (Figure 8B), termed FAM110D+ OS, UBE2C+ OS, CAVIN1+ OS and ZFAS1+ OS, each characterized by their respective marker genes as illustrated in Figure 8C,D. Biologically, FAM110D+ OS was primarily implicated in the extracellular matrix, cell adhesion and ossification, suggesting a propensity for tissue residency (Figure 8E). UBE2C+ OS, apart from mediating its own cell adhesion, also facilitated the adhesion of leukocytes and the Ras signalling transduction, indicating a predisposition towards regulating immune cell functions and possessing a higher proliferative potential (Figure 8E). ZFAS1+ OS was also mainly involved in regulating immune cell functions, while CAVIN1+ OS tended towards mediating protein modifications and responding to external stressors (Figure 8E).

Mitophagy phenotype analyses revealed that, in comparison to other malignant osteoblasts, mitophagy was significantly upregulated in FAM110D+ OS and UBE2C+ OS, with the latter exhibiting the most pronounced upregulation (Figure 8F–H). Both cell types were involved in tissue residency, immune cell regulation and proliferative control. Further stemness analysis indicated that these two cell types shared similar stemness levels, suggesting a parallel ecological niche in the cellular differentiation trajectory (Figure 8I,J). Consequently, UBE2C+ OS, characterized by the most significant upregulation of mitophagy, had its biological functions further elucidated. GSEA quantitatively identified the biological processes upregulated in UBE2C+ OS. As shown in Figure 3, UBE2C+ OS selectively upregulated various immune processes, including T cell differentiation, T cell selection and also enhanced the proliferative pathways associated with osteoblasts (Figure 8K). Moreover, transfer learning suggested a broadly similar cell cycle distribution between UBE2C+ OS and FAM110D+ OS (Figure 8L,M). Collectively, malignant osteoblastic cells, by upregulating mitophagy, interfered with immune cell adhesion, T cell differentiation and subgroup selection, thereby endowing malignant osteoblastic cells with a higher proliferative potential.

### 3.8 Mitophagy affected malignant osteoblasts in early‐middle developmental phase

**FIGURE 9:**
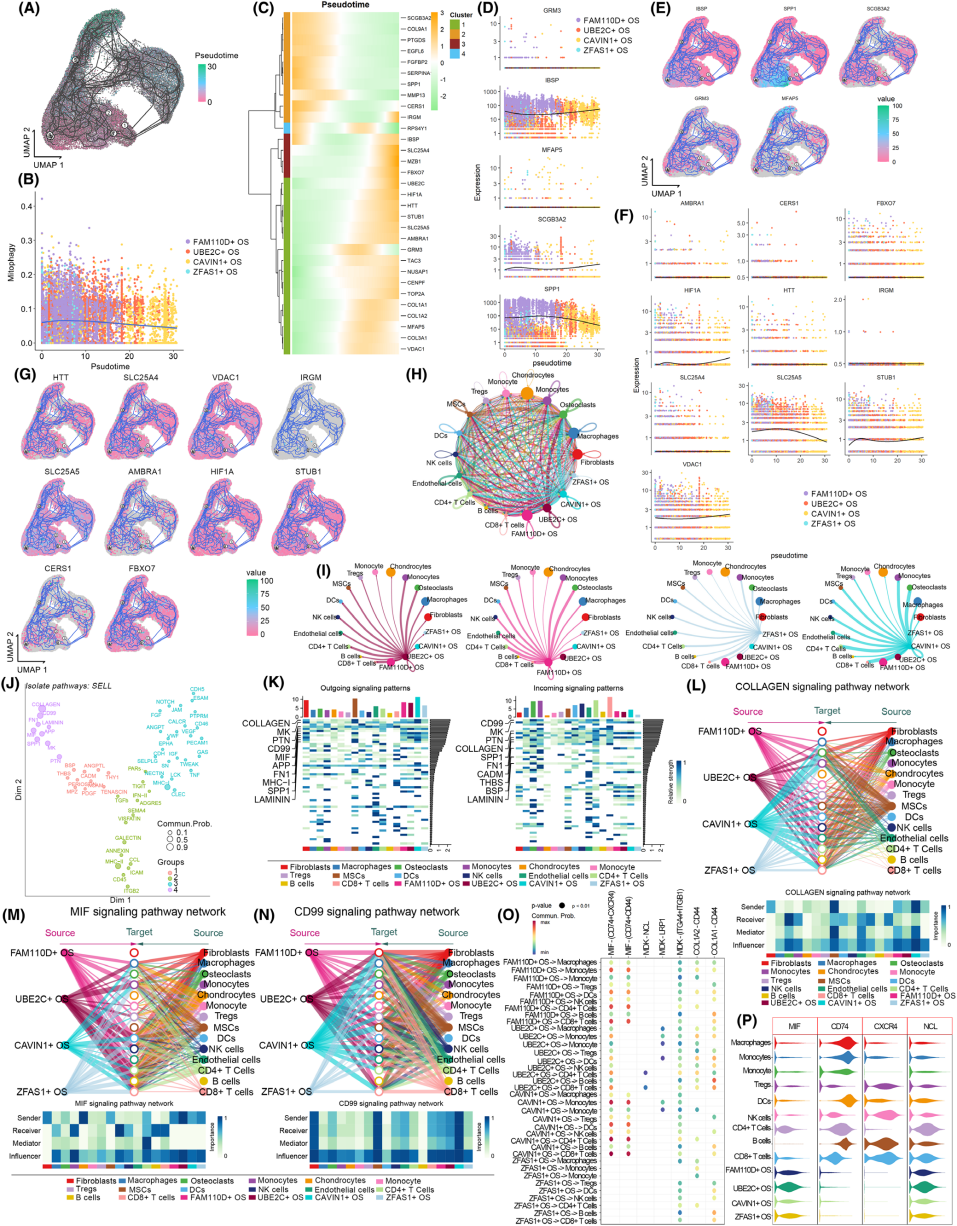
Cell development analyses and cell–cell communication. (A) The developmental lineage of malignant osteoblasts. (B) Alterations of mitophagy during malignant osteoblasts' development. (C) The gene expression patterns' alteration during development. (D) and (E) Top variable genes during development. (F, G) The alterations of mitophagy genes during development. (H) The overall cell–cell communication. (I) The cell–cell communication of different malignant osteoblasts. (J) The identified cell–cell communication patterns. (K) The overall outgoing and incoming signalling patterns of osteosarcoma cells. (L–N) Collagen, MIF and CD99 signalling. (O, P) Ligand‐receptor analyses.

The developmental evolution of tumour cells determined their malignancy. Therefore, CytoTRACE was employed to dissect the differentiation potential of malignant osteoblasts. As resultant data detailed in Figure 8N,O, FAM110D+ OS and UBE2C+ OS exhibited the highest differentiation potential, suggesting their positioning in the early to mid‐stages of malignant osteoblast development. In contrast, CAVIN1+ OS demonstrated the lowest differentiation potential, indicating its potential placement at the terminus of cellular development. Subsequently, cell developmental trajectory tracking was conducted to elucidate the impact of mitophagy on the development of malignant osteoblasts. Malignant osteoblasts primarily originate from FAM110D+ OS and ZFAS1+ OS, subsequently developing into UBE2C+ OS, and ultimately differentiating into CAVIN1+ OS, consistent with the cell differentiation potential analysis (Figure 9A). UBE2C+ OS may represent a de‐differentiation developmental trend. Consistent with aforementioned findings, mitophagy was consistently moderately upregulated (Figure 9B); however, its upregulation was most pronounced during the early to mid‐stages of malignant osteoblast development, indicating a selective upregulation of mitophagy by malignant osteoblasts during these stages. The developmental trajectory of malignant osteoblasts led to four distinctly different gene expression patterns (Figure 9C); among them, the top five genes with the most significant changes in expression as development progressed were shown in Figure 9D,E. The trend of changes in mitophagy pathway genes was depicted in Figure 9F,G.

### 3.9 High‐mitophagy malignant osteoblasts regulated B cells, DCs, CD8+ T cells, NK cells via MIF‐(CD74 + CXCR4)‐mediated MIF signalling

Analyses of mitophagy in malignant osteoblast subtypes revealed that overactivation of mitophagy in FAM110D+ OS and UBE2C+ OS regulated their interactions with immune cells and other malignant cells; hence, cell–cell communication analyses were conducted to elucidate mitophagy‐mediated intercellular interactions. As detailed in Figure 9H, the interactions among stromal cells, immune cells and malignant cells within osteosarcomas were exceedingly complex and highly intense. Malignant osteoblast subtypes exhibited significant interactions with immune cells such as CD8+ T cells, CD4+ T cells and DCs to varying extents (Figure 9I). Specifically, the overall cell communication within osteosarcomas exhibited four patterns, mediating osteogenesis, immunity, inflammation and proliferation (Figure 9J). Outgoing and incoming signalling pattern analyses indicated that signalings such as Collagen, MK, MIF, PTN and CD99 dominated the intercellular communication modes among cells within osteosarcomas (Figure 9K). Notably, the four malignant osteogenic cell subtypes, especially those upregulating mitophagy, FAM110D+ OS and UBE2C+ OS, were found to dispatch signals, including MIF, CD99 and Collagen, that could potentially influence the behaviour of various immune cells (Figure 9L–N). For example, activation of MIF signalling by UBE2C+ OS and FAM110D+ OS might regulate B cells, DCs, CD8+ T cells, NK cells and monocytes (Figure 9M).

Receptor–ligand analysis was employed to further decipher the potential communication between malignant osteoblasts and immune cells. As detailed in Figure 9O,P, malignant osteoblasts, particularly UBE2C+ OS with the most significant upregulation of mitophagy, by overexpressing MIF, potentially modulated B cells, DCs, CD8+ T cells, NK cells, etc., through MIF‐(CD74 + CXCR4) receptor–ligand interactions. Therefore, while these findings suggested a potential pathway by which mitophagy and MIF signalling could mediate interactions between malignant osteoblasts and various immune cells, further experimental validation was necessary to confirm these mechanistic links and their biological significance.

## 4 DISCUSSION

The current investigation, to our knowledge, constitutes the first endeavour to develop a well‐designed deep‐learning framework aimed at the identification of pivotal genes governing mitophagy, thereby enabling the advancement of mitophagy‐centric therapeutic strategies to counteract drug resistance. BayeDEM, upon application to osteosarcoma datasets, demonstrated unparalleled accuracy in modelling the correlation between transcriptomic data and the mitophagy phenotype, identifying CERS1 as a critical gene regulating mitophagy in osteosarcoma. More heartening, predicated on CERS1 identified through BayeDEM, this study has successfully devised and validated in preclinical cell models, a combinatory treatment regimen against CDDP‐resistant osteosarcoma. This regimen operates by inhibiting CERS1 to attenuate mitophagy, thus compromising the mitochondrial quality control mechanisms in CDDP‐resistant osteosarcoma cells, rendering them susceptible to CDDP‐induced apoptosis. Mechanistically, mitophagy predominantly impacts malignant osteoblasts during early to mid‐development stages, especially those expressing UBE2C and FAM110D. It significantly upregulates pathways associated with osteoblast proliferation and actively engages in antigen presentation, T‐cell differentiation and other immune signal transductions. Immunologically, malignant osteoblasts with high mitophagy levels mediate the MIF signalling transmission between malignant osteoblasts and B cells, DCs, CD8+ T cells, NK cells and monocytes through the MIF‐(CD74 + CXCR4) receptor–ligand interaction, thereby modulating the biological functions of these immune cells.

Benefiting from the exponential growth of cancer sequencing data, deep learning has gradually emerged as the preferred tool for identifying cancer phenotypes, predicting patient survival risk and identifying key genes in critical biological processes of tumours.^9^, ^33^, ^34^ Real‐world tumour sequencing data exhibit high‐dimensional characteristics that typically do not conform to a normal distribution; even after various normalization treatments, the data remain a mixture of several distributions and cannot be described by a standalone distribution.^9^ Hence, traditional statistical models struggle to fit the data distribution in such high‐dimensional spaces with mixed data relationships, leading to their suboptimal performance.^9^ In contrast, deep learning theoretically could fit any data distribution and could handle high‐dimensional data through well‐designed feature selection modules. For example, pipelines constructed by integrating different deep learning models could predict the resistance of different human cancers to various drugs with high precision, significantly facilitating the pre‐treatment decisions of clinical physicians.^9^ Additionally, different deep learning frameworks could be used for the subtyping of various malignancies with accuracy comparable to that of experienced clinical physicians,^35^, ^36^ thereby simplifying the preliminary diagnosis of tumours. This inspiring evidences prompted the development of BayeDEM, designed to process ultra‐high‐dimensional transcriptomic data and precisely identify key genes regulating mitophagy. BayeDEM achieved 100% AUC of the PR curve and 98% AUC of the ROC curve in the test set, indicating that BayeDEM could fit the association between transcriptomic data and mitophagy with extremely high precision and discrimination. The exceptional performance of BayeDEM is attributed to its built‐in Bayesian optimizer, which models historical observations sequentially and efficiently searches for the optimal combination of hyperparameters, thereby enabling BayeDEM to achieve optimal performance. Similarly, previous investigations have employed the Monte Carlo Tree Search (MCTS) methodology for the refinement of deep learning architectures, aiming at forecasting the responsiveness of patients to chemotherapeutic agents.^9^ In contrast to MCTS, the merits of Tree‐structured Parzen Estimator (TPE) predominantly encompass enhanced efficiency in navigating high‐dimensional domains, accelerated convergence towards solutions in specific optimization challenges—particularly those characterized by continuous or conditional hyperparameters—and a generally reduced computational demand relative to the exhaustive exploration intrinsic to MCTS. TPE demonstrates exceptional aptitude for the optimization of hyperparameters within the realm of machine learning, adeptly pinpointing areas within the search space warranting further investigation. Potentially, BayeDEM could be used to identify key genes regulating mitophagy in any tumour, thus paving the way for oncologists to study mitophagy.

Ceramide, a pivotal constituent of sphingomyelin within the cellular phospholipid bilayer,^37^ is intricately involved in the dysregulation observed in a multitude of human pathologies.^38^, ^39^ The genesis of ceramide predominantly arises from cellular catabolic processes, with ceramide synthase (CERS) playing a critical role in the re‐acylation of sphingosine.^40^ CESR1, a member of the CERS family, has been implicated in the malignant progression of various carcinomas, including those of the head, neck and lungs.^40^, ^41^ Moreover, CERS1 appears to be linked to lysosome‐mediated autophagy.^42^ This study represents the inaugural confirmation that CERS1 is a critical regulator of mitophagy in osteosarcoma, with its overexpression leading to the hyperactivation of mitophagy. Mitophagy, a quintessential mechanism for mitochondrial quality control, preserves the mitochondrial pool by eliminating damaged mitochondria, thereby averting apoptosis.^2^ Furthermore, this investigation corroborates that heightened mitophagy is associated with an adverse prognosis in osteosarcoma. CDDP, a frontline chemotherapeutic agent for osteosarcoma, impedes tumour progression by inducing apoptosis in osteosarcoma cells, whereas the upregulation of mitophagy can counteract the mitochondrial damage and subsequent apoptosis induced by it. Consequently, this research pioneers a novel combinatorial drug strategy aimed at mitigating CDDP resistance in osteosarcoma cells by inhibiting CERS1. The administration of a CERS1 inhibitor was found to resensitize CDDP‐resistant osteosarcoma cells to CDDP, inhibiting their proliferation, migration, invasion and inducing apoptosis. Mechanistically, the inhibition of CERS1 disrupts mitochondrial quality control in CDDP‐resistant osteosarcoma cells through the inhibition of mitophagy, leading to the dissipation of mitochondrial membrane potential and unfavourable alterations in mitochondrial dynamics. Therefore, this study posits that targeting CERS1 to combat CDDP resistance in osteosarcoma may represent a promising therapeutic strategy.

The dynamic interplay between tumour cells and immune cells orchestrates the formation and evolution of the tumour‐specific microenvironment, encompassing the inactivation of cytotoxic immune cell functions, the establishment of an immunosuppressive microenvironment and the differentiation of pro‐tumorigenic immune cell subtypes.^43^ Moreover, metabolic reprogramming, recognized as one of the hallmarks of cancer, is intricately linked to tumour immunity; mitophagy serves as a crucial mechanism in this context by regulating mitochondrial quality control to participate in cancer metabolic reprogramming.^44^ Consistent with this evidence, our study reveals that osteosarcoma subtypes with high mitophagy significantly modulate T‐cell differentiation, adhesion and antigen presentation, indicating that mitophagy plays a pivotal role in regulating the immunosuppressive microenvironment of osteosarcoma. The macrophage migration inhibitory factor (MIF) signalling pathway, which is dysregulated to varying extents across all types of immune cells, is a key mediator by which tumour cells regulate immune cells.^45^ MIF has been proven to inhibit various immune cell activities, including cell infiltration, maturation and antigen presentation. In neuroblastoma, MIF mediates the suppression of macrophage migration and T‐cell responses.^46^ Through single‐cell level intercellular communication analysis, our study discovered that mitophagy mediates the MIF signalling between malignant osteoblasts and B cells, DCs, CD8+ T cells, NK cells and monocytes via the MIF‐(CD74 + CXCR4) receptor–ligand interaction, thereby modulating the biological functions of these immune cells. Collectively, the active regulation of various immune cells by mitophagy culminates in the immunosuppressive microenvironment observed in osteosarcoma.

The limitations of the present study must be acknowledged. Deep learning models, including the present BayeDEM, have overfitting risk and generalizability concerns. Further cross‐validation by including an external cohort is beneficial to address such issues.

## AUTHOR CONTRIBUTIONS

**Wenyi Jin:** Conceptualization (equal); data curation (equal); formal analysis (equal); investigation (equal); methodology (equal); validation (equal); visualization (equal); writing – original draft (equal); writing – review and editing (equal). **Junwen Chen:** Data curation (equal); formal analysis (equal); investigation (equal); software (equal); validation (equal); visualization (equal); writing – original draft (equal). **Zhongyi Li:** Data curation (equal); formal analysis (equal); investigation (equal); methodology (equal); writing – original draft (equal). **Zhang Yubiao:** Methodology (equal); project administration (equal); validation (equal); writing – review and editing (equal). **Hao Peng:** Funding acquisition (equal); project administration (equal); supervision (equal); writing – review and editing (equal).

## FUNDING INFORMATION

This study was funded by the National Natural Science Foundation of China (No. 81672154) and the Hubei Provincial Key Research and Development Program (2021BCA147).

## CONFLICT OF INTEREST STATEMENT

The authors declare that the research was conducted in the absence of any commercial or financial relationships that could be construed as a potential conflict of interest.

## Supporting information


Table S1.

Figure S1.

## Data Availability

The original contributions presented in the study are included in the article/Supplementary Material. Further inquiries can be directed to the corresponding authors.
