# Supplementary figures and images for: Bayesian‐optimized deep learning for identifying essential genes of mitophagy and fostering therapies to combat drug resistance in human cancers
Source: J Cell Mol Med. 2025 Jan 21;29(2):e18254. doi: 10.1111/jcmm.18254 (PMC11747347; doi:10.1111/jcmm.18254)

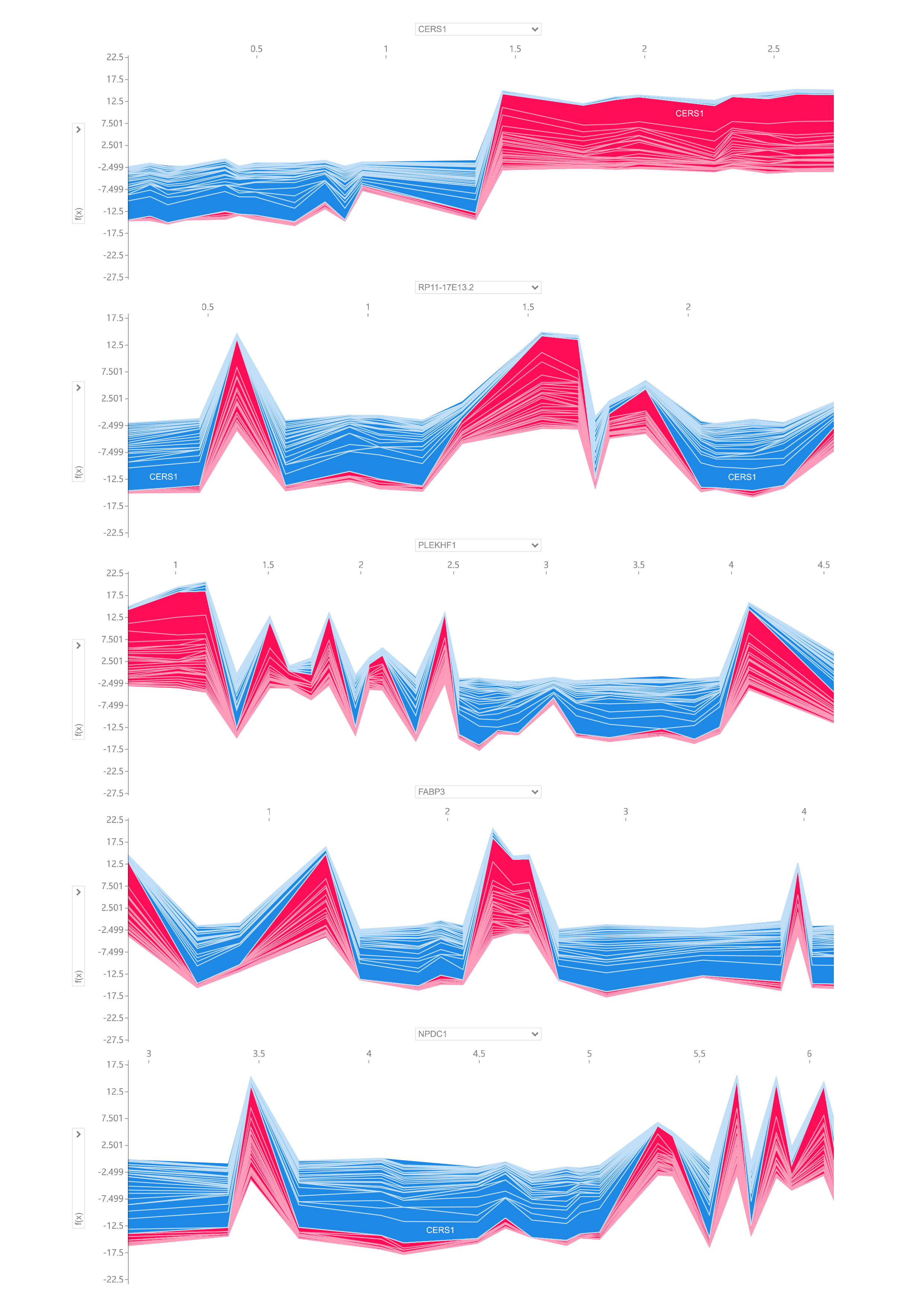

Supplement: Supplementary file 2 — Figure S1. [file JCMM-29-e18254-s001.tif]
